# Index Air Quality Monitoring for Light and Active Mobility

**DOI:** 10.3390/s24103170

**Published:** 2024-05-16

**Authors:** Stefano Botticini, Elisabetta Comini, Salvatore Dello Iacono, Alessandra Flammini, Luigi Gaioni, Andrea Galliani, Luca Ghislotti, Paolo Lazzaroni, Valerio Re, Emiliano Sisinni, Matteo Verzeroli, Dario Zappa

**Affiliations:** 1 Department of Information Engineering, University of Brescia, 25123 Brescia, Italy; stefano.botticini@unibs.it (S.B.); elisabetta.comini@unibs.it (E.C.); emiliano.sisinni@unibs.it (E.S.); dario.zappa@unibs.it (D.Z.); 2 Department of Engineering and Applied Science, University of Bergamo, 24129 Bergamo, Italy; luigi.gaioni@unibg.it (L.G.); andrea.galliani@unibg.it (A.G.); luca.ghislotti@unibg.it (L.G.); paolo.lazzaroni@unibg.it (P.L.); valerio.re@unibg.it (V.R.); matteo.verzeroli@unibg.it (M.V.)

**Keywords:** light mobility, air quality index, MOx sensors

## Abstract

Light and active mobility, as well as multimodal mobility, could significantly contribute to decarbonization. Air quality is a key parameter to monitor the environment in terms of health and leisure benefits. In a possible scenario, wearables and recharge stations could supply information about a distributed monitoring system of air quality. The availability of low-power, smart, low-cost, compact embedded systems, such as Arduino Nicla Sense ME, based on BME688 by Bosch, Reutlingen, Germany, and powered by suitable software tools, can provide the hardware to be easily integrated into wearables as well as in solar-powered EVSE (Electric Vehicle Supply Equipment) for scooters and e-bikes. In this way, each e-vehicle, bike, or EVSE can contribute to a distributed monitoring network providing real-time information about micro-climate and pollution. This work experimentally investigates the capability of the BME688 environmental sensor to provide useful and detailed information about air quality. Initial experimental results from measurements in non-controlled and controlled environments show that BME688 is suited to detect the human-perceived air quality. CO_2_ readout can also be significant for other gas (e.g., CO), while IAQ (Index for Air Quality, from 0 to 500) is heavily affected by relative humidity, and its significance below 250 is quite low for an outdoor uncontrolled environment.

## 1. Introduction

In an era marked by continuously increasing concerns over climate change and human well-being, and where sustainable living is intimately connected to technological advancements, initiatives promoting active mobility and environment-aware solutions stand at the forefront of innovation. Among these initiatives, the MOST project (Centro Nazionale per la Mobilità Sostenibile. Available online: https://www.centronazionalemost.it/ (accessed on 1 May 2024)) funded by the Italian PNRR (Piano Nazionale di Ripresa e Resilienza) and part of the Next Generation EU program, aims at the development of innovative solutions that will help, on the one hand, to create a system of safe, accessible, and accident-free mobility and, on the other hand, to promote green, clean, and climate-neutral mobility habits. Within the scope of the project, the development of low-cost, low-power solutions for the monitoring of environmental parameters crucial for human well-being is a key topic.

Air quality remains a critical public health concern, both indoors and outdoors, with far-reaching consequences for the global population. Recent estimates from the World Health Organization (WHO) reveal that air pollution contributes to millions of premature deaths annually, highlighting its substantial impact [1]. Nearly the entire global population breathes air exceeding WHO air quality guidelines [2], putting it at risk for different health problems. Sensors are key devices in the development of cost-effective monitoring systems for air quality. The number of sensors deployed worldwide is forecast to reach the one trillion mark by 2025 [3]; hence, nowadays, there exists a wide variety of commercially available sensor devices deployed in buildings for environmental monitoring purposes; these devices employ different technologies and functionalities (such as temperature, carbon dioxide, humidity, occupancy, light, and airflow sensors), use a wide range of communication techniques, wired and wireless (such as Ethernet, Power Line Carrier, Zigbee, Bluetooth, Wifi, EnOcean, BACnet, Modbus, LoWPAN, Z-Wave, LoRaWAN, etc.), and sometimes can also be powered with batteries and energy harvesting sources. In addition, the right placement of the sensors in the environment is of crucial importance for the optimal functioning of the system.

Over the past few years, miniaturized metal oxide (MOx) sensors have gained significant popularity for monitoring air quality [4]. Such sensors, also referred to as chemiresistive sensors, offer a broad range of detection capabilities for different harmful gasses, with a device’s resistance being affected by the exposure to both reducing and oxidizing gasses. There are several low-cost and compact MOx sensors, as described in Section 3. An interesting, well-known, easily available, integrated, and low-power solution is the BME680/688 sensor from Bosch SensorTec, capable of detecting the presence of volatile compounds (VOCs) in indoor air, excluding CO_2_. These devices natively furnish an estimation of VOC concentration and CO_2_ concentration in parts per million and also compute a comprehensive relative air quality index, and, in the range 0 ≤ IAQ ≤ 500. According to the manufacturer, for IAQ ≤ 50, air quality is good, while IAQ ≥ 200 identifies polluted air.

In this work, an experimental characterization of the performance of the Bosch BME688 sensor is discussed, with the final aim of evaluating whether this sensor, mainly conceived for HVAC applications, can be successfully exploited for low-cost, low-power solutions for environmental monitoring in light and smart mobility applications. The conducted experiments are mainly focused on the effects of temperature and humidity and the dependence of the estimated values from environmental conditions. The validity of the results can be used not only to design new, sensorized tools for e-bikes, such as cycling accessories or charging stations, but also for a more general cooperative, distributed environmental monitoring, including agriculture and animal husbandry.

Starting from a brief review of air pollutants and how current literature and directives interpret the air quality in Section 2, Section 3 reports the main technologies for air quality monitoring with a particular focus on the Bosch SensorTec BME688 and the Arduino Nicla Sense ME. Section 4 describes the three case studies and experimental setups, while Section 6 discusses the results obtained from the measurement campaigns carried out with the proposed demonstrator. The final section draws some conclusions on the possibility of using BME688 for environmental monitoring.

## 2. Air Quality

Clean air mainly consists of 78% nitrogen, 21% oxygen, and a mix of different gasses such as carbon dioxide, argon, and hydrogen [5]. The WHO distinguishes two groups of pollutants: the first one includes primary pollutants, which are directly emitted into the atmosphere, like sulfur dioxide (SO_2_), nitrogen oxides (NOxs), carbon monoxide (CO), volatile organic compounds (VOCs), and particulate matter (PM). The second group includes the so-called secondary pollutants, which are the result of chemical reactions of primary pollutants, such as ozone (O_3_), some nitrogen compounds, and secondary PM. Measuring the secondary pollutants is more difficult because their formation depends on environmental conditions, such as chemical precursors, temperature, and sunlight intensity. In urban areas, people can be exposed to large concentrations of primary pollutants, with levels of exposure being influenced by the means of transport [6,7]. For cyclists and pedestrians, the amount of inhaled pollutants is higher due to an increased breathing rate [8]. Chronic exposure to air pollutants has been linked to several adverse health outcomes, ranging from respiratory to cardiovascular diseases, and it has been shown to exacerbate existing conditions such as asthma and allergies [9].

The detrimental effects of air pollutants disproportionately affect the most vulnerable in our society, including children, elders, and individuals with pre-existing health conditions. Hence, monitoring outdoor air quality is crucial for tackling local environmental issues and protecting public health. To achieve this, the European Union delivered several regulations, including the Ambient Air Directive (2008/50/EC) [10], also referred to as the “EU Clean Air Directive”, which outlines different measures that member states must put in place to monitor and improve air quality. Amongst these, the directive sets the minimum number of fixed air quality monitoring stations (AQMSs) needed in different regions. These stations are strategically located based on air pollution levels, population density, and coverage area. The AQMS leverages reliable and certified methods to measure different pollutants. However, the initial investment and ongoing maintenance costs for these stations can be significant and may limit their usage. Advancements in sensor technology offer new solutions to air quality monitoring: such solutions promise a more cost-effective way to gather data but come with the trade-off of lower accuracy and reliability compared to traditional AQMSs. Despite this, the large availability of low-cost sensors enables high-resolution and real-time data collection, offering valuable insights into air quality trends and fluctuations. While not as precise as traditional monitoring stations, many studies demonstrate that these systems can be effectively used to assess air quality, providing a general understanding of pollution levels. As a significant example, the project described in [11] is based on a network of eleven sensor nodes (ten stationary and one mobile unit mounted on a public bus) spread across the city of Bari, Italy. Such a network successfully measured various pollutants like NO_2_, O_3_, CO, and PM10, together with environmental parameters such as temperature and humidity, using electrochemical and optical sensors. The results described in the study revealed a high success rate in classifying outdoor air quality, based on the air quality index (AQI), compared to the results from the closest official AQMS station.

Assessing the air quality index serves as a robust and informative method for communicating the outdoor air quality conditions across a given region. The computation of the AQI leverages different methodologies depending on the reference air quality standards. Among these methodologies, the Environmental Protection Agency’s (EPA) AQI stands as one of the most widely recognized frameworks, strictly linked with the National Ambient Air Quality Standards (NAAQS) table (United Stated Environmental Protection Agency (EPA) “NAAQS Table”. Available online: https://www.epa.gov/criteria-air-pollutants/naaqs-table (accessed on 8 May 2024)). Such a table serves as a reference point laying out concentration limits for six principal air pollutants deemed critical to public health and environmental integrity. These pollutants include carbon monoxide, lead, nitrogen dioxide, ozone, particulate matter (PM10 and PM2.5), and sulfur dioxide. By establishing specific concentration limits for each of these pollutants, the NAAQS provides a framework for assessing air quality and formulating mitigation strategies to safeguard public health and ecological balance. The air quality index is a numerical value ranging from 0 to 500: an AQI of 100 is typically associated with healthy air quality for most people.

Together with the assessment of outdoor air quality, indoor air quality is of uttermost importance for increasing the quality of life. High-quality indoor air must satisfy the requirements of low CO_2_ and VOCs levels, and comfortable air temperature and humidity; not complying with these requests can lead to Sick Building Syndrome (SBS), a situation where a relationship between the time spent in a specific building and a negative impact on health can be observed [12]. Numerous indoor pollutants have well-documented detrimental impacts on human health; these range from causing irritation in the nasal and mucous membranes to potentially leading to permanent damage or even cancer. Interestingly, elevated levels of CO_2_ in indoor air have been found to have a statistically significant correlation with declines in cognitive performance and this holds true even at levels that are considered acceptable according to standards such as ASHRAE [13]. Additionally, volatile organic compounds (VOCs) can result in the irritation of the ears, nose, and throat, while particulate matter can contribute to the development of lung diseases. Usually, building ventilation is achieved in a natural way, by diluting polluted indoor air with fresh and outdoor air, or by cleaning and reusing the indoor air; however, the first method, where the ventilation rate is controlled by opening and closing windows, can reliably ensure good air quality only locally, near the window, and will increase heating systems’ energy consumption [12]. The main purpose of HVAC (heating, ventilation, and air conditioning) systems is to provide a comfortable, healthy, and productive indoor environment responsive to humans and their demands [14], and their employment contributes significantly to the overall energy consumption of modern buildings. It is estimated that building energy consumption accounts for 30–45% of the global energy demand [3], where the largest component comes from heating and cooling; in fact, in [15] the authors state that above 60–70% of the primary energy used in major parts of the world such as the US, EU, China, and Australia is intended for this purpose. Given the enormous energy consumption of HVAC systems, one of the key aspects to achieve their optimal usage is an accurate sensing and measurement of various parameters such as temperature, humidity, pressure, and air quality. The characterization of sensors used in HVAC systems and their synergetic use is crucial for ensuring optimal performance, energy efficiency, and indoor air quality (IAQ); by improving fault diagnosis and control strategies, significant savings can be achieved. Many national and international agencies suggest and recommend limit values for main pollutants, both outdoors and indoors. Table 1 shows the exposure limit values for some gasses according to different agencies and organizations.

## 3. Sensors for Air Quality Evaluation

Among the different sensors integrated in monitoring systems, temperature, humidity, and gas sensors are key devices that strongly impact the performance of these systems. Temperature sensors measure the temperature of a flowing media such as air, water, etc. [3] and are an essential element in modern HVAC systems. Generally, there are four types of temperature sensors: thermocouples, RTDs (resistance temperature detectors), thermistors, and IC sensors, each with different properties and features, in terms of ease of operation, accuracy, range of measurement, response time, and cost.

Humidity sensors quantify the amount of water vapor in air, which is typically measured in terms of relative humidity (measured as a function of temperature), dew point (measured as a function of pressure of the gas), or absolute humidity. The functioning of these devices typically relies on two fundamental physical operating principles, namely the high-performance capacitive one and the low-cost resistive one. In the first case, metallic plates (usually gold or platinum) and dielectric material (usually a plastic or polymer) get deposited onto a substrate (glass, silicon, or alumina) to obtain an equivalent capacitor; humidity levels of the surrounding air affect the capacitance, which gets measured to obtain the dielectric constant, proportional to this level. These sensors present a high level of linearity, enabling them to accurately measure relative humidity (RH) in the range 0–100%. They exhibit exceptional accuracy, within a range from ±0.1% to ±5.0%, and are known for their low power consumption; nevertheless, capacitive sensors necessitate intricate circuitry, regular calibration, and are comparatively more expensive than alternative humidity sensor technologies. Conversely, resistive sensors are, on average, low-cost, possess a good response time (10–60 s), and tend to have a narrow measurement range (usually unable to measure values lower than 5% RH) and less accuracy (±1–10%) [3].

Carbon dioxide sensors facilitate the supervision and regulation of carbon levels to guarantee safety and uphold high indoor AQI, while reducing unnecessary air changes that lead to higher energy usage. Optical gas sensors leverage the principle of optical absorption, described by the Beer–Lambert law [22], to measure the concentration of carbon dioxide. This technology relies on the property of CO_2_ molecules of absorbing infrared light featuring a wavelength close to 4.2 μm. By measuring the amount of absorbed light and comparing it with a known concentration, the CO_2_ concentration in a gas sample can be assessed. This technology, known as transmissive NDIR (non-dispersive infrared), requires a minimum optical path length of several centimeters to ensure sufficient absorption of infrared rays at low CO_2_ concentrations [23]. NDIR sensors have high accuracy (±30–200 ppm), wide measuring range (0–10,000 ppm), durability, reliability, slow response times (30–100 s), and high power consumption [3]. As a robust alternative to transmissive NDIR sensors, the photo-acoustic NDIR technology, also known as Photo-Acoustic Spectroscopy (PAS), allows for smaller sizes and enhanced robustness against mechanical and thermal stress [24]. PAS technology is based on the measurement of the intensity of pressure waves associated with the vibrations of CO_2_ molecules, which, in turn, are the results of infrared light absorption by the molecules themselves. By measuring the amplitude of the pressure waves with a microphone inside a measurement chamber, the CO_2_ concentration can be calculated. As an example, the Infineon PASCO2V01 [25] leverages PAS technology to provide highly sensitive and accurate carbon dioxide detection. Such a sensor is designed to measure CO_2_ concentrations in different applications, including indoor air quality monitoring, HVAC systems, and automotive and industrial processes.

Other air pollutants, like CO and volatile organic compounds (VOCs), are commonly detected with metal oxide semiconductor (MOx)-based and electrochemical-based sensors. Electrochemical gas sensors are usually exploited for detecting inorganic gasses such as CO, NO_2_, SO_2_, and O_3_, providing a change in the current across two electrodes related to the concentration of a target gas, chemically reacting with the sensing membrane. They exhibit response times from 10 s to 60 s, an accuracy of ±30 ppm, measurement ranges from 0 to 1000 ppm, higher sensitivities, longer life spans, and lower energy consumption when compared with MOx sensors [3]. As an example, TGS5141-P00 by Figaro [26] and the AQ7CO by Honeywell [27] can be successfully exploited to measure the concentration of carbon monoxide in the air, despite being powered by a battery. In the realm of air quality sensing, phosphorene is gaining attention for its remarkable chemical, physical, and optoelectronic characteristics. Combined with the capabilities of standard air quality sensors, phosphorene, with its unique properties, may lead to more sensitive, selective, and efficient sensing technologies [28].

The use of miniaturized MOx sensors became popular for monitoring air quality [17] because they offer a broad range of detection capabilities for harmful gasses. The MOx sensor structure includes a thermally insulated heating membrane fabricated through silicon micromachining techniques, which enables the production of smaller sensors with lower power consumption. A metal oxide layer is placed on top of the heating membrane. This layer is made of a doped semiconductor material, such as stannic oxide (SnO_2_), tungsten trioxide (WO_3_), or titanium dioxide (TiO_2_). At high temperatures, gas molecules react with the surface of the MOx material, generating free charge carriers that induce a resistance variation in the material. Oxidizing gasses, such as NOx, increase the resistance of the sensor, whereas reducing gasses, like VOCs, lead to a decrease in the resistance. It has been previously shown that changes in MOx sensor electrical responses, which undergo this band-bending phenomenon, can be influenced by temperature and humidity [9]. To account for these influences, many sensor assemblies, including the one targeted in this study, incorporate additional circuitry. These devices have a VOC measurement accuracy that can vary from ±30 ppm up to ±100 ppm in a wide measurement range (400–20,000 ppm) and response time from 50 s to 60 s. Energy consumptions are usually high, as they require an electric heater to maintain the sensing element temperature at up to 300 °C, and present short life spans [3]. As an example, the ZMOD4410 sensor by Renesas [29,30] and the ENS160 by ScioSense [31] provide the VOC concentration in parts per billion (ppb) and, at the same time, calculate the corresponding air quality level based on the UBA standard.

In [32], authors reviewed CMOS Micro-Electro-Mechanical Systems (MEMSs) for gas detection, sensor technologies, design, and operation in the field of indoor air quality control and monitoring, with a focus on commercially available products. In [33], an extensive summary of a series of modular IoT platforms and gas sensor nodes for real-time monitoring of the AQI is provided, and a flexible IoT (Internet of Things) gas sensor node, using a modular functional concept for the fast detection of small leakages and hazardous gas situations, is presented. Among the most common MOx n-type sensors in this field of application, the literature mentions the *BME680* (SnO_2_ [34]) and BME688 from Bosch SensorTec [35], the SGP30 (MOx coated nano-particles [34]) from Sensirion, and IAQ-Core [33] and CCS811 [34] from AMS. The BME680 sensor is a digital device that detects the presence of volatile compounds in indoor air, excluding CO_2_. It is capable of estimating the total amount of VOCs in the surrounding air and measuring temperature, humidity, and pressure. On the other hand, the CCS811 sensor is an energy-efficient digital gas sensor specifically designed to monitor indoor air quality. It processes the raw sensor data and provides temperature, TVOC measurements in parts per billion (ppb), and an estimated CO_2_ equivalent in parts per million (ppm). Lastly, the SGP30 sensor is the first metal oxide gas sensor to incorporate multiple sensors on a single chip. It also provides a TVOC measurement in parts per billion (ppb), and an equivalent CO_2_ concentration in parts per million (ppm). It is worth noting that each of these metal oxide sensors includes on-chip algorithms to adjust the TVOC output based on humidity and temperature. BME688 and SPG30 are gas sensors that come as tiny digital solutions that already handle the heater control, calibration procedures, baseline and long-term correction, and humidity compensation (for BME688, partially supported by a related processing library), and offer a comfortable interface such as SPI or I2C [33]. In [36], a comparison among many popular MOx sensors has been reported; however, the study neither consider the impact of AI on the estimation of air pollutants and AIQ, nor firmware-configurable devices like the ZMOD4410 by Renesas and the software supported BME688. For the time being, the BME688 sensor is the only solution combining precise environmental sensing with AI capabilities, the latter being easily upgradable thanks to the tools provided by the vendor. This, together with the large availability of breakout boards and the Nicla environment, as well as additional devices such as IMUs, make it an ideal candidate for the development of lightweight mobile applications. In Table 2, a brief comparison between a subset of properties of commercially available air quality or pollutant sensors is provided as a summary of the previous discussion.

In this work, the BME688 was considered as a reference example of an MOx sensor. The performance of the BME688 sensor is discussed, with the aim of evaluating whether this sensor, mainly conceived for HVAC applications, can be successfully exploited for low-cost, low-power solutions for environmental monitoring. The choice of configuring the BME688 sensor using the Nicla Sense board was led by the advanced functionalities made available in the rather compact size of the device. This, together with the low-power features of the platform, shown in Table 2, make it one of the best candidates in view of the development of portable equipment to be installed in light vehicles such as e-bikes. Moreover, it has to be noted that the Nicla Sense board is fully compatible with other Arduino products, making it possible to easily integrate the device into existing systems, leveraging the power of the Arduino ecosystem. Moreover, the BME688 sensor’s capabilities open doors for other interesting possibilities in different fields. As an example, in smart homes and buildings, the BME688 can monitor air quality, eventually triggering ventilation systems or generating alerts, ultimately promoting a healthier indoor environment. In industrial settings, such a device can be exploited to detect harmful gas leaks or build-up, improving worker safety and ensuring compliance with environmental regulations.

The Nicla Sense ME was selected for this study due to its availability from many different suppliers and distributors, and for its wide acceptance in academia. As a matter of fact, many different works in the literature designed proof of concepts around this board. As an example, in [37], the Nicla Sense ME was used to gather environmental parameters for an alert system capable of warning drivers of possible attention decrease; in [38], it was used as the reference sensing platform in a fog-like computing architecture for industrial applications. In the field of environmental parameters monitoring, the Nicla Sense ME was used in [39] as a real-time monitoring solution in sleeping environments in order to improve sleep quality. Moreover, this platform has been proposed as a personal environmental device with the aim of providing a cost-effective personal environmental exposure monitoring device evaluating the detrimental effects of pollutants (by measuring the levels of CO, CO_2_, O_3_, H_2_S, as well as temperature, pressure, motion, and the relative concentration of VOCs) on human health [40]. However, to the best of authors’ knowledge, just a few works in the literature report systematic measurement data relevant to the BME688 sensor [33,41]. Nonetheless, it should be noted that such works focus on the behavior of the BME688 exposed to VOCs such as hexane and ethanol, while this paper is mainly concerned with the study of CO_2_ concentration and of the IAQ index, together with raw data made available from the sensor including the effects of temperature and relative humidity.

## 4. The Adopted Demonstrator

A demonstrator was devised to acquire data in the previously described environment. The main components in the system are presented in the following section, starting with the MOx sensor under investigation, namely the Bosch BME688.

### 4.1. The Bosh SensorTec BME688

The BME688 from Bosch SensorTec offers a combination of environmental- and gas-sensing capabilities within a single, compact integrated circuit (IC). It is housed in a 3.0 × 3.0 × 0.93 mm metal lid Land Grid Array (LGA) package. It can operate in a temperature range from −40 °C to +85 °C, with a relative humidity level in the range from 0% to 100% and pressures varying from 300 mbar to 1100 mbar. As reported in Section 1, MOx sensors, beside cross-sensitivity with temperature, are mainly dependent on humidity, a parameter which should be kept under control while operating the device. For this purpose, the sensor integrates additional on-board environmental-sensing devices, including temperature, humidity, and pressure as reported in the Bosch SensorTec BME688 datasheet [35].

The BME688 represents an improved version of the BME680, where gas scanning functions have been introduced leveraging configurable artificial intelligence (AI) models, although the AI functionality is external to the device [42]. Bosch BME688 and its older counterpart BME680 provide a relative air quality index from 0 to 500, where values larger than 200 reflect a condition of polluted air. These sensors also provide an estimation of VOC concentration in parts per million, tested on a mixture of elements typically exhaled by human respiration.

The temperature sensor integrated within the chip utilizes a micromachined thermopile [43] composed of a number of serially connected thermocouples. Each thermocouple consists of two dissimilar metals (p-type and n-type doped silicon) that generate a Seebeck voltage proportional to the temperature difference across their junction due to the Seebeck effect. The multiple thermocouples are connected electrically in series and thermally in parallel, resulting in an amplified output voltage. The amplified voltage is then processed by on-chip amplification and signal conditioning circuitry. Subsequently, an analog-to-digital converter (ADC) within the IC converts the conditioned signal into a high-resolution digital temperature output. The absolute accuracy of the temperature sensor between 0 °C and 65 °C is ±0.5 °C.

A humidity sensor is also available in the package. It is machined as interdigitated electrodes fabricated on a hygroscopic dielectric layer. The capacitance between the electrodes is dependent on the permittivity of the dielectric layer, which varies with the adsorbed water vapor molecules. The BME688 incorporates an on-chip oscillator circuit to measure the capacitance [44]. The oscillation frequency is inversely proportional to the capacitance. Therefore, changes in humidity induce a shift in the oscillation frequency, which is converted into a digital output using a frequency-to-digital converter (FDC) within the IC. The response time of the humidity sensor, evaluated as the response of a humidity change varying as a step function and quite difficult to be experimentally evaluated, is 8 s at 63% of the steady-state value. The relative humidity accuracy is ±3% with a hysteresis of ±1.5%.

Completing the environmental sensor lot, a pressure sensor is present in the IC. It leverages a piezoresistive pressure transducer comprising a thin, flexible membrane made of a piezoresistive material. When exposed to pressure, the membrane experiences a deflection, causing a change in its electrical resistance due to the piezoresistive effect. The BME688 employs a Wheatstone bridge [45] circuit to measure this resistance change. The bridge becomes unbalanced due to the altered resistance, resulting in a voltage difference at the output terminals. This voltage difference is amplified and processed by the on-chip electronics, providing a digital pressure output proportional to the applied pressure. The Root Mean Square (RMS) noise is 0.12 Pa, with an offset temperature coefficient of ±1.3 Pa/K.

**Figure 1:**
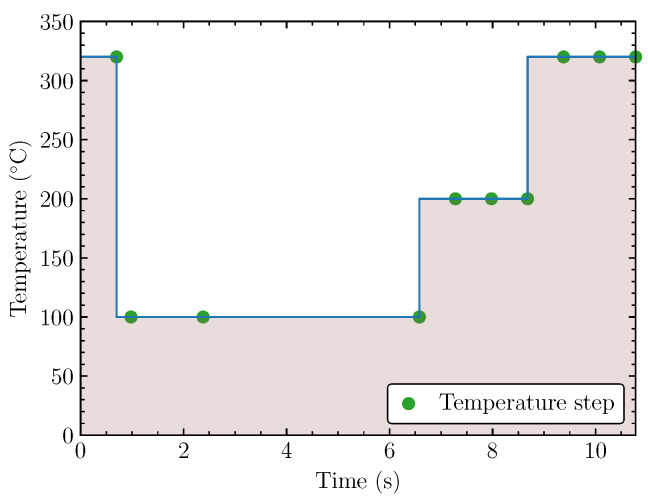
BME688 default heating profile, adapted from [35].

The MOx sensor comprises a metal oxide layer deposited on a ceramic substrate. This metal oxide exhibits semiconducting behavior, and its electrical conductivity is influenced by the presence of various gasses in the surrounding environment. As discussed in Section 1, the interaction between gas molecules and the metal oxide surface leads to charge transfer processes, altering the resistance and current flow through the sensor. However, the MOx sensor response is often complex and non-specific. The gas sensor incorporates an integrated micro-hotplate heater [46], enabling the use of heating profiles (Bosch Sensortec “BME AI Studio Documentation—Glossary”. Available online: https://www.bosch-sensortec.com/software/bme/docs/overview/glossary.html#measurement-session (accessed on 8 May 2024)) to improve gas-sensing capabilities. These profiles represent a set of controlled temperature cycles applied to the sensor, influencing its sensitivity and selectivity towards specific gaseous compounds. The standard heater profile is provided in Figure 1. Selectivity in gas sensing refers to the sensor’s ability to distinguish between different gas species present in a mixture and respond primarily to the target gas of interest.

The primary purpose of the heating profile is to enhance the BME688’s sensitivity to gas molecules, particularly volatile organic compounds (VOCs) [47]. Heating the sensor element increases the desorption of gas molecules adsorbed on the sensing surface, leading to a stronger signal and improved detection limits. This is achieved through Temperature-Programmed Desorption (TPD) facilitated by the heating profile [48]. When heated, gas molecules adsorbed on the sensor surface desorb, meaning that they are released back into the gas phase. However, the desorption process is temperature-dependent: different gas molecules exhibit varying desorption temperatures. Some of them are readily desorbed at lower temperatures, while others require higher temperatures for efficient desorption. Furthermore, the heating profile can be customized to improve selectivity by exploiting the temperature-dependent desorption characteristics of different gas species. By adjusting the temperature and duration of the heating cycles, the sensor can be made more responsive to specific target gasses while minimizing interference from other environmental factors that might influence the readings. By tailoring the temperature and duration of the heating profile, the BME688 can selectively target the desorption of specific gas molecules.

### 4.2. Bosch SensorTec Development Environment

In order to retrieve higher-level information from the raw data extracted by the BME688, Bosch SensorTec developed a library called Bosch Software Environmental Cluster (BSEC) (Available online: https://www.bosch-sensortec.com/software-tools/software/bme680-software-bsec/ (accessed on 7 May 2024)) to be used by the microcontroller that reads the sensor. The BSEC library is a part of the BSEC system, which is composed of BME68x sensors, sensor driver Application Programming Interfaces (APIs), the BSEC fusion library, and the BME AI Studio. By suitably combining raw data from the physical sensor, the BSEC module makes it possible to shape virtual sensors with enhanced capabilities. The sensor driver APIs are useful software interfaces that handle the low-level interactions with the sensor. They provide the possibility to retrieve compensated raw data via the I2C or SPI communication bus. The BME AI Studio serves as an AI toolchain designed for the development of personalized classification and regression AI models.

The BSEC library can be customized according to the desired application through a configuration file. This file includes information concerning the supply voltage, the different power modes, and the number of days considered for the automatic background calibration of the air quality index. These parameters influence the processing, the output data, and they can affect the performance of the library overall. The supply voltage of the sensor can be selected between two different values, 1.8 V and 3.3 V, and it influences the self-heating of the sensor. Since the BME688 can be used in different scenarios, there exist multiple modes of operation that differ in power consumption and output data rate. The gas scan mode (SCAN) is designed for applying regression and classification algorithms on targeted gasses. The BSEC algorithm has been trained to identify the presence of H_2_S and to estimate its concentration in ppm. In this use case, the standard heater profile is used, with an update rate of 10.8 s. Different heater profiles, classification, and regression algorithms can be designed with the BME AI Studio. Continuous mode (CONT), low-power mode (LP), and ultra-low-power mode (ULP) provide some interesting output data, such as IAQ, estimated CO_2_, and breath-VOC equivalent concentration with an output rate of 1 s, 3 s and 300 s, respectively. They also have different average power consumptions: less than 12 mA for the continuous mode, less than 1 mA, and 0.1 mA for the low-power and ultra-low-power modes. Finally, it is possible to choose the number of days for the automatic background calibration for the IAQ: the possible choices are 4 days or 28 days. The output given by the BSEC library depends on the selected mode, except for the raw data relevant to temperature, humidity, pressure, and gas resistance which can be retrieved in all modes. In scan mode, the BSEC provides the probability of estimation and the regression result for at most four gas classes. On the other hand, in CONT, LP, and ULP, it is possible to get IAQ and static-IAQ (s-IAQ) values. These indexes can assume a value from 0 to 500 and they give insight about the air quality. As shown in Table 3, values above 100 mean polluted air.

Another virtual sensor provided by the BSEC is the equivalent CO_2_, which relates to the concentration of the CO_2_ leveraging the correlation between the VOC and CO_2_ exhaled by humans. The BSEC library also provides the possibility to get an estimation of the corresponding concentration of the VOC in referring to a breath-VOC gas mixture tested in the laboratory. Finally, it provides some heater-compensated temperature and humidity values which give more accurate temperature and humidity values.

### 4.3. Arduino Nicla Sense ME Board

In the context of the proposed setup, as discussed in the following section, a BME688 breakout board was needed to minimize wiring related to power and communication. The Arduino Nicla Sense ME board (Arduino “Documentation for *Arduino Nicla Sense ME*”. Available online: https://docs.arduino.cc/hardware/nicla-sense-me/ (accessed on 7 May 2024)) comes in handy in this scenario, providing both a BME688 sensor, additional sensors, and integrating Bluetooth functionalities in its microcontroller. The Arduino Nicla Sense ME is a development board with networking capabilities designed to evaluate wireless sensor network architectures, powered by an on-board sensor fusion engine. For this purpose, the board integrates the BHI260AP sensor hub [49], a pressure sensor, a three-axis magnetometer, and the BME688 directly connected to the sensor hub.

The sensor hub IC acts as a hardware accelerator equipped with a programmable 32-bit microcontroller, a six-axis Inertial Measurement Unit (IMU), and an event-driven software framework with data processing capabilities (Bosch Sensortec “Sensor Fusion Software (BSX)”. Available online: https://www.bosch-sensortec.com/software-tools/software/sensor-fusion-software-bsx/ (accessed on 7 May 2024)). This chip has 25 GPIO and standard communication interfaces (SPI/I2C). The BHI260AP is responsible for reading and processing the data from the sensors connected to it, lightening the computational load of the central control unit (host) to which it is connected. When programmed with proper firmware, the sensor hub is able to manage complex algorithms, aggregating data from multiple sensors by creating virtual sensor objects. This chip aims to provide a computational subsystem and hardware centralizer for devices that may need to process huge amounts of data for prolonged periods with low power consumption. It can also be used as a smart sensor for orientation, motion recognition, and activity recognition, thanks to the six degrees of freedom IMU that it integrates.

**Figure 2:**
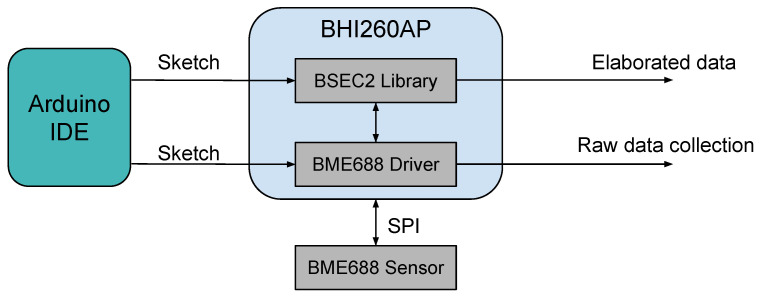
Diagram representing the firmware configuration scheme of BME688 through Arduino IDE.

The sensor hub can be equipped with drivers that enable the dialogue with the sensors, setting their operating parameters and receiving their readings. The Arduino Nicla Sense ME is compatible with firmware that must be flashed specifically through the Arduino IDE and that manages the sensor hub configurations. The firmware loaded in the BHI260AP device, when configured through the Arduino IDE interface, employs the Sensorboard Default HP/RDC configuration, which means that the HP-354 and the standard RDC are used. The BSEC library runs on the BHI260AP’s built-in processing unit. The BME688 sensor hardware needs to be configured with proper operational settings. These settings need to be configured on the BME688 sensor HW through the BHI260AP on the Nicla Sense ME board, and this can be done through the Arduino BHY2’s library APIs. The Nicla Sense ME configuration diagram with its sensor hub firmware and the BME688 data management is reported in the diagram of Figure 2.

The Nicla Sense ME board is powered by an nRF52832 System on Chip (SoC) within the ANNA-B112 module; this means that the Bluetooth Low Energy (BLE) module is available. The sensor hub and the microcontroller exchange information using the SPI protocol, which, in turn, exchange information with the BME688 using the same protocol. It is possible to read data from the BME688 and send it to a receiving wireless device via BLE or a UART.

## 5. Experimental Setup

This work experimentally investigates the capability of the BME688 environmental sensor to provide useful and detailed information about air quality. For this purpose, three experimental setups have been designed to perform measurements in free and controlled environments.

The Arduino Nicla Sense ME boards have been programmed to output, through Bluetooth or UART, the following data:

- Raw value of temperature in degrees Celsius, T [°C];
- Raw value of relative humidity, RH [%];
- Raw value of pressure, P [mbar];
- Raw value of the sensor resistance, gas resistance [kΩ];
- Estimated value of CO_2_, CO_2eq_ [ppm];
- Index of air quality by Bosch, in arbitrary units, from 0 to 500, IAQ.

### 5.1. Office Space Experimental Setup

As shown in the introduction, the BME688’s main application destination is HVAC, and monitoring polluted air generated by breath. In order to verify the performance of the BME688 sensor, a very simple, indoor experimental setup was considered. Two Nicla Sense ME boards were left in a 50 $m^{3}$ office for about one month, from 22 December 2023 at 10 a.m. to 20 January 2024 at 14 p.m. (29 days, 4 h).

With the exception of the first eight hours, the office HVAC was turned off, and the office was closed for 263 h, from the start of the experiment to 2 January 2024 at 8 a.m. One person entered the office after 483 h from the start of the experiment, staying in the office for about 10 h, that is from 11 January 2024 at about 8 a.m. and leaving the office at about 18 p.m. Two people entered the office after 569 h from the start of the experiment, staying for about 10 h, that is from 15 January 2024 at about 8 a.m. and leaving the office at about 18 p.m. This was repeated from Monday to Friday.

### 5.2. Climatic Chamber Setup

The behavior of the BME688 sensor was evaluated under controlled environment conditions. To achieve this, the ACS DY110 climatic chamber, a programmable test chamber specifically designed by ACS (Angelantoni Test Technologies “ACS: Camere per prove ambientali simulate dal 1952”. Available online: https://www.acstestchambers.com/it/ (accessed on 11 April 2024)) for precise thermal characterization of small components, was used.

**Figure 3:**
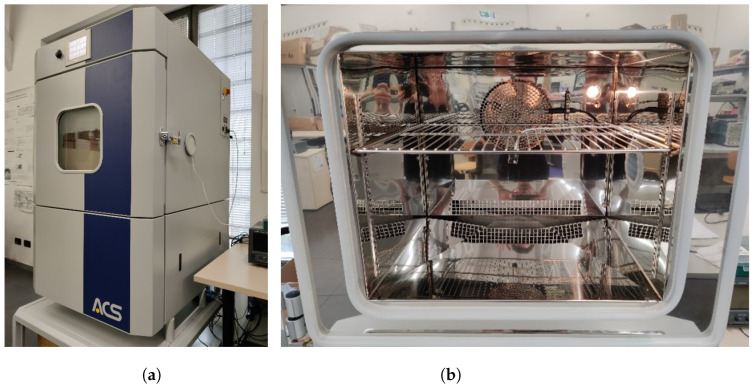
The ACS DY110 test chamber adopted for the characterization of the BME866 sensor (**a**) and the Nicla Sense ME placed inside the active volume of the chamber (**b**).

The DY110 chamber, shown in Figure 3a, offers a capacity of 110 L, and features external dimensions of approximately 877 × 1080 × 1434 mm. A crucial feature of the instrument is the precise temperature control, in a range from −40 °C to +180 °C with rather small fluctuations, typically not exceeding ±0.3 °C. Heating and cooling rates are close to 3 °C min^−1^ and 4 °C min^−1^, respectively. For applications requiring combined temperature and humidity control, the DY110 test chamber is able to set humidity levels between 10% and 95%, with fluctuations generally smaller than ±3%. Such a wide range can be particularly useful for simulating real-world environments that encompass both temperature and humidity variations. By leveraging the capabilities of the DY110 climatic chamber, valuable insights into the BME688 sensor’s accuracy and stability can be gained.

The Nicla Sense ME board was placed hanging on the grid frame inside the chamber, as in Figure 3b, specifically at the center of the inner volume of the chamber, in order not to suffer from excessive ventilation due to the compressor or very small gradients of temperature and humidity at the edges of the working volume. In this setup, the board was powered via USB, exploiting the chamber’s lateral porthole, while data were read through Bluetooth. The Data Acquisition (DAQ) system was purposely devised using a Raspberry Pi 3+, which managed and monitored the BLE connection to the board inside the chamber. This single-board controller offers off-the-shelf functionalities and modules that comprehend, but are not limited to, WiFi and Bluetooth connections, as well as a fully functional, customizable Linux-based OS system. Such a device was preferred over others for the ability to use high-level programming languages for the acquisition suite, so as to enable fast development and deployment, given the large availability of libraries dealing with low-level communication and data processing.

**Figure 4:**
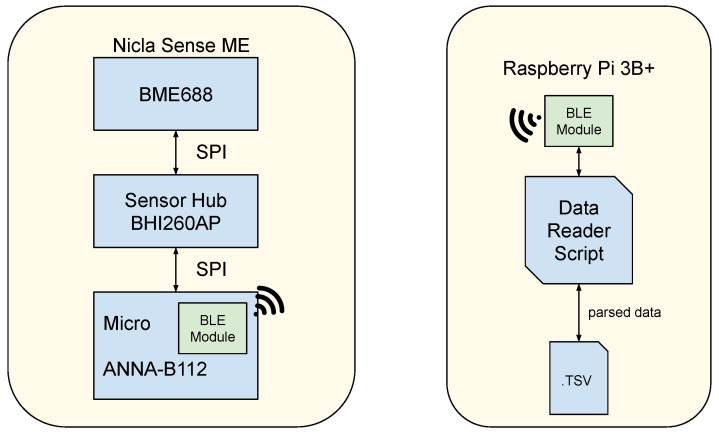
Demonstrator information flow. The devices and the integrated components are highlighted.

The demonstrator architecture is reported in Figure 4. The Nicla Sense ME board gathers data from each sensor mounted on the board through requests managed by the sensor hub. A unique timestamp is associated with the record of measured values. The DAQ draws from the meaningful services provided by the Bluetooth Low Energy (BLE) peripheral, the Nicla Sense ME board, and logs them in a local database. The software is not only responsible for getting and logging the data but also for ensuring that these are consistent in terms of format and frequency of acquisition and tunable to the needs of the experiment. Furthermore, it ensures that the Bluetooth connection is maintained or recovered if lost. The readout data are stored properly in a file containing, in each row, the record acquired through the communication, comprising the timestamp, temperature, humidity, and gas resistance data.

### 5.3. Fluxing Chamber Setup

The setup consisted of a sealed custom stainless-steel chamber (1 liter volume) located inside a climatic chamber (MPM Instruments s.r.l “Incubatore refrigerato-Refrigerating incubator”. Available online: http://www.mpminstruments.com/Tbr.htm (accessed on 11 April 2024)) with a PID thermo-regulator, able to maintain the inner temperature in a 5.0–80.0 °C range with ±0.5 °C stability. The use of a climatic chamber allowed the performance of measurements at different ambient temperatures, regardless of the room temperature. In particular, measurements were performed at 10, 20, and 30 °C.

Humidified air was produced by synthetic dry air through a Drechsel bottle, held in a thermostatic bath (MPM Instruments s.r.l “Bagno refrigerato - Refrigerator thermostatic bath.” Available online: http://www.mpminstruments.com/Bmr.htm (accessed on 11 April 2024)) at 5 °C higher than the selected ambient temperature, and then in a condensation vessel in order to favor the condensation of saturated vapor. The thermostatic bath had a temperature range from 5.0 to 99.9 °C, with an accuracy of ±0.5 °C at 37 °C. The humidified air was then mixed with synthetic dry air in order to obtain the desired relative humidity (RH) content from 0% to 80%. The mass flow controllers adopted for the fluxing setup (MKS Instruments “MKS 1179C01352CR1BV Mass Flow Controller”. Available online: https://www.mks.com/p/1179C01352CR1BV (accessed on 2 May 2024)) mixed the different gasses, maintaining the total flow of 200 SCCM inside the test chamber. These mass flow controllers had a control range of 2–100% with an accuracy of ±1.0% of the full scale. Two Nicla Sense ME boards were placed inside the stainless-steel fluxing chamber; however, due to the presence of two metallic sealed enclosures, the DAQ system previously described was not effective in this setup. For this reason, a custom board was used in order to provide power and mechanically support the two Niclas. An externally placed laboratory power supply provided power to the system (Thurlby Thandar instruments “PL330DP-DC Power Supply”. Datasheet available online: https://www.sglabs.it/public/TTI_PL330DP.pdf (accessed on 1 May 2024)); meanwhile, the devices were programmed to transmit the acquired data via a serial connection (UART). To overcome noise disturbances from other equipment, RS-232 levels and adequate circuitry were used to transmit data. As previously described, data were collected by means of a Raspberry Pi and stored in a plain text file.

**Figure 5:**
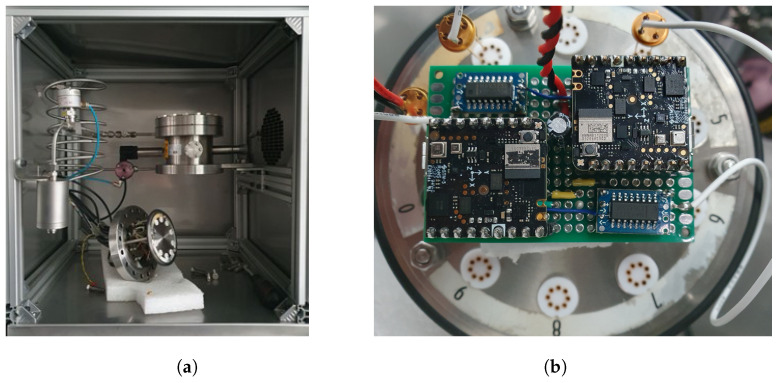
Fluxing chamber setup. Custom stainless-steel chamber (1 L volume) located inside M120-TBR climatic chamber (**a**) and details of the connection of the Nicla Sense ME to metallic transistor outline (**b**).

A picture of the fluxing-chamber setup is shown in Figure 5. Figure 5a shows the interior of the climatic chamber with the custom 1 L volume stainless-steel chamber opened and divided into two parts: the fixed chamber and the removable part (on the bottom) with transistor carriers and a sealed BNC connection. Figure 5b shows the custom carrier board for the two Nicla Sense ME; it is possible to see the two Nicla Sense ME boards and the serial transceiver (blue boards).

We used a flow-through technique to investigate the response of the BME688 sensor, as reported in a previous study [50]. After positioning the custom board with the two Nicla, connecting the wires, and closing the stainless-steel chamber, several tests were performed to assess the behavior of the system. Initially, three tests were performed at fixed temperatures of 10, 20, and 30 °C, respectively, to verify if, with the rise in relative humidity, the IAQ indicator, and CO_2_ estimation continued to be reliable and stayed within acceptable thresholds; different levels of controlled relative humidity were achieved for each test, resulting in the following profile: (a) the board was turned on in presence of a humid air flow of 200 SCCM at a relative humidity of 20% for 2 h, (b) relative humidity was then increased to 40% for 1 h, (c) relative humidity was further raised to 70% for 1 h, (d) relative humidity was then reduced back to 40% for 1 h, and (e) relative humidity was again decreased to 20% for 2 h. Each test had a total duration of 7 h.

Two other tests were performed at a temperature of 20 °C and relative humidity of 20% in order to verify Nicla’s outputs in the presence of CO and CO_2_, respectively. Test gasses with a certified composition, supplied by SOL S.p.A. (“SOL GROUP-Technical and Medical Gases”. Available online: https://www.sol.it/it (accessed on 11 April 2024)), were mixed in a carrier of dry synthetic air by mass flow controllers, maintaining the total flow of 200 SCCM. Gas concentrations of various analytes (CO and CO_2_) were chosen based on a possible application of BME688 for CO_2_ monitoring in indoor environments. For each concentration, gas was injected in the pipeline for 5 min but not in the test chamber to remove any trace of previous gasses. Afterward, gas was injected into the chamber for 30 min, and then the synthetic air was restored for 1 h. CO_2_ and CO gas concentrations during injections into the chamber were 600 ppm and 20 ppm, respectively; these values were selected based on the guidelines described by the WHO. In indoor environments, CO_2_ levels are often used as an indicator of occupancy due to the fact that humans exhale CO_2_ when they breathe; when carbon dioxide levels exceed 600 ppm, it is typically interpreted as a sign of occupancy within the area. This threshold is frequently exploited as a standard for ensuring satisfactory indoor air quality in a range of environments, such as workplaces, educational facilities, and communal areas. Conversely, CO is not a byproduct of human activity, but rather a result of the incomplete combustion of fuels containing carbon; a presence of 20 ppm of CO in the atmosphere may suggest the proximity of combustion sources. Although this concentration is deemed low, it is crucial to address it promptly, as prolonged exposure to even minimal levels of carbon monoxide can lead to negative health consequences.

## 6. Experimental Results

This section shows and discusses the results of the measurements in the experimental setups described in the previous section.

### 6.1. Tests in Office

Two Devices Under Test (DUTs), namely Nicla_1_ and Nicla_2_, were placed in an indoor environment as described in the previous section. All the available raw measures plus the estimated CO_2_ and IAQ of the two devices were acquired and stored in order to be further post-processed.

**Figure 6:**
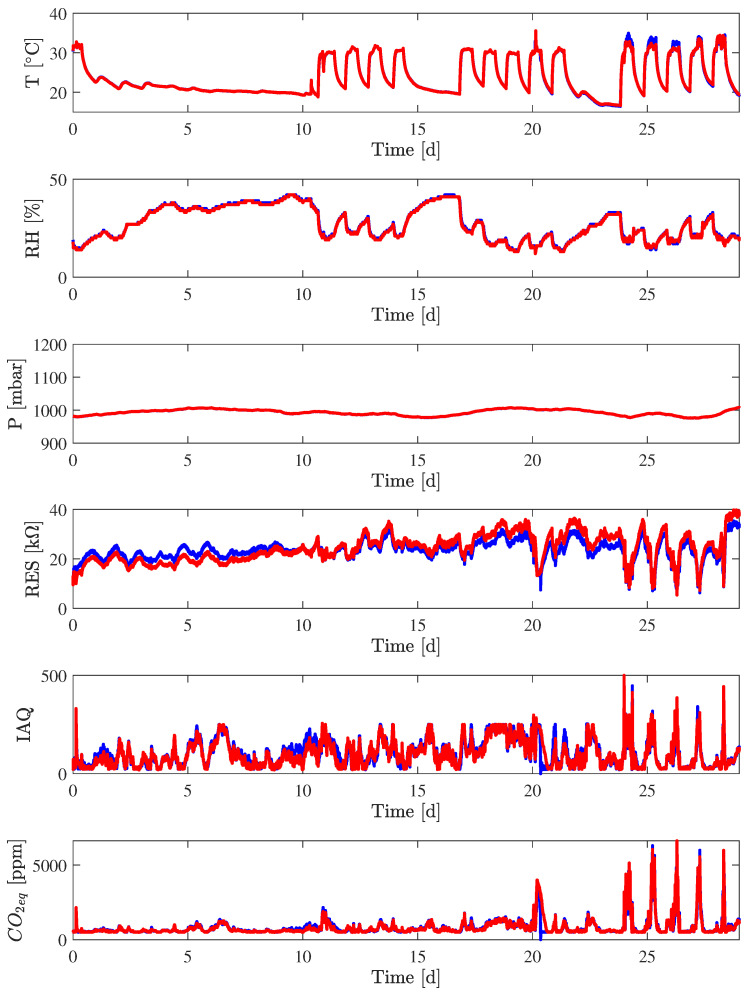
Acquired measurements (T, RH, P, RES, CO_2eq_, and IAQ) of the experiments in the office space for the Nicla_1_ (in blue) and Nicla_2_ (in red).

Figure 6 shows the behavior for Nicla_1_ and Nicla_2_ for temperature (T) in degrees Celsius, relative humidity (RH) in percentage, pressure (P) in mbar, resistance of sensor (RES) in kΩ, estimated CO_2_ in ppm and the index of air quality (IAQ) by Bosch, in arbitrary units (a.u.) from 0 to 500 with a threshold at about 250. As it is possible to see from the picture, the measurements made by the two devices show a very good agreement. This behavior is also reported in Table 4, where the statistical values (mean value μ, standard deviation σ, and maximum excursion Δ) for the measured quantities T, RH, and P of the previous acquisition are reported. The GAS resistance correlation between Nicla_1_ and Nicla_2_ is 0.92, while it reaches 0.95 and 0.93 for CO_2eq_ and IAQ, respectively. It should be noted that estimated CO_2_ is limited to below 500 ppm (default value), which is close to the value of outdoor air (400–500 ppm). The value of IAQ, whose lower limit is 25, corresponding to the output value during the sensor initialization phase, reports a saturation around 250 if there are no people in the room; that is, the CO_2eq_ is below a few thousand parts per million. T and RH are raw, uncompensated data; simple linear compensation can be done to achieve the correct results. It should be noted that when comparing the measured temperature results with a sensor Sensirion SHT31, an offset of about 7 °C is present.

The estimated peak value of CO_2_ concentration of about 4000 ppm with one person in the office occurred at day 20 and partially at day 21, and a concentration of about 6000 ppm with two persons was registered from days from 24 to 28; these measurements are in accordance with the expected value due to the presence of two persons in the room. The air conditioning system gets air from outdoors, where the expected CO_2_ concentration is typically 400 ppm (Data taken from NOOA Global Monitoring Laboratory “Trends in CO_2_, CH_4_, N_2_O, SF_6_—Carbon Cycle Greenhouse Gases”. Available online: https://gml.noaa.gov/ccgg/trends/global.html (accessed on 2 May 2024)). One person at rest produces about 500 L daily of CO_2_ (Brian Palmer “Do We Exhale Carbon?” Available online: https://www.nrdc.org/stories/do-we-exhale-carbon (accessed on 8 May 2024)); so, in about 8 h, the production is about 165 L, and in an office of about 50 m^3^, it is about 3300 ppm, with an overall value of about 3700 ppm.

The approximately one-month worth of experimental results were divided into three logic portions: when the office is not air-conditioned and empty, when the office is air-conditioned and empty, and when the office is air-conditioned and occupied. In order to obtain an indication of the strength of the linear relationship among each couple of measured quantities, the Pearson linear correlation coefficient [51] $\rho_{xy}$ was calculated as (1):

(1) $$\rho_{xy}=\frac{\sum_{k}\left(x\left[k\right]-\mu_{x}\right)\left(y\left[k\right]-\mu_{y}\right)}{\sigma_{x}\sigma_{y}},$$

where $\mu_{x}$ and $\mu_{y}$ are the mean values of the generic signals x[k] and y[k], while $\sigma_{x}$ and $\sigma_{y}$ are the standard deviations of the measures. In Table 5, the correlation $\rho_{xy}$ is calculated for the Nicla_1_ measurements limited from 26 December 2023 at 00:00 to 2 January 2024 at 00:00, exactly one week, during which the office was empty and not air-conditioned.

This is the case that is the most similar to the outdoor environment.

The correlation reported in Table 6 refers to days 2nd–5th and 8th–10th of January from 9 a.m. to 7 p.m., with the office air-conditioned but empty.

The correlation reported in Table 7 is related to days 11th, 12th, and 15th–19th January, with the office air-conditioned and occupied.

The three tables show a high correlation between the estimated CO_2_ and IAQ, of about 90%, and a high correlation between RH and P, of more than 80%, similar to outdoor conditions. Without air conditioning and without people adding CO_2_ and VOCs, the gas resistance shows a high correlation with temperature, of more than 75%, while this value decreases to below 30% in the presence of air conditioning. In the presence of air conditioning but without persons, the gas resistance shows a high correlation with relative humidity, while, in the presence of persons, the correlation with CO_2_ is dominant.

### 6.2. Tests in Climatic Chamber

**Figure 7:**
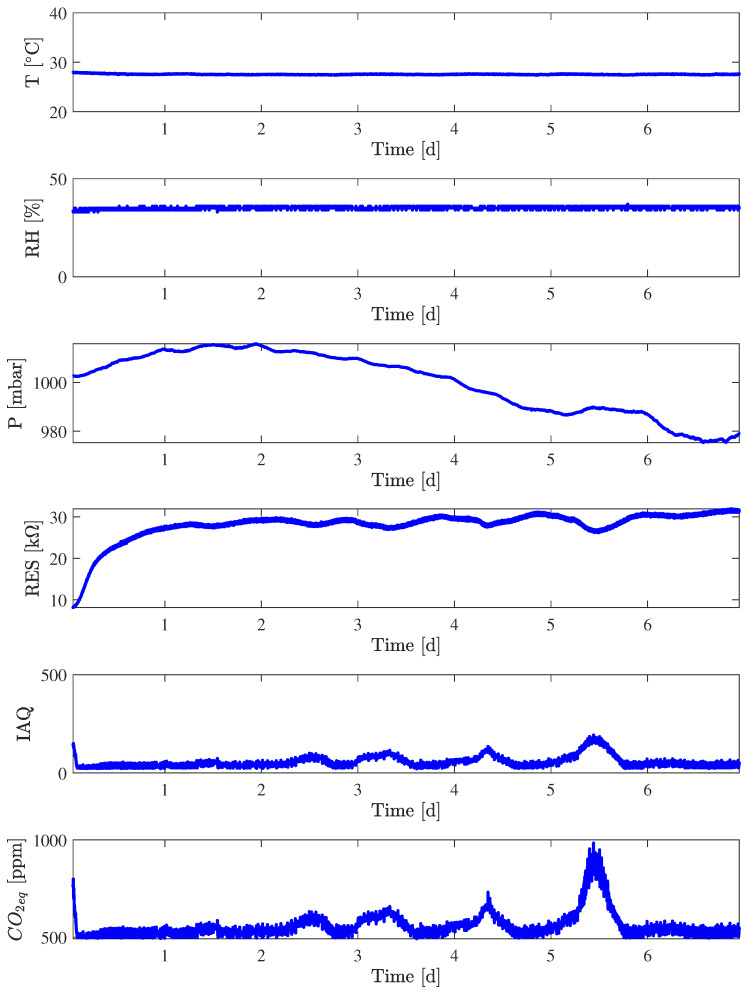
Measures (T, RH, P, RES, and CO_2eq_, IAQ) acquired in the climatic chamber for Nicla_3_.

The data collection campaign inside the chamber lasted about one week, from Friday 15 December 2023 at 11:10 a.m. to Friday 22 December 2023 at 13:45 p.m. with a constant temperature of 25.00 (3) °C and a constant relative humidity of 35.00 (33)% set by the climatic chamber itself. The values sent by the Nicla Sense ME board are reported in Figure 7. It is worth noting that the Nicla Sense ME employed during this experiment (Nicla_3_) is a different one from the two used in the previous experiments. The first two days (16 December 2023 and 17 December 2023) were a Saturday and a Sunday, days in which the air conditioning system of the environment external to the chamber was off, while the rest of the days (from 18 December 2023 to 12 December 2023) were normal working days, with normal air conditioning. A more marked variation of gas resistance can be ascribed to both air conditioning status and people’s presence during working days.

Starting from the raw value of temperature, Nicla_3_ reports a relatively stable reading of 27.55 (6) °C over the whole experiment time, after an initial settling which reports higher temperatures. There is an underlying periodicity in the temperature measurements, limited within 2 °C, more marked from the third day onward, of about 24 h. Another important factor to highlight is the offset from the actual average temperature climatic chamber set-point, with the average temperature reading of the Nicla_3_ of about +2.55 °C, an effect which is expected in sensors, which needs to be properly calibrated. The RH reading is very stable across the week of measurement, with variations due to sensor resolution.

**Figure 8:**
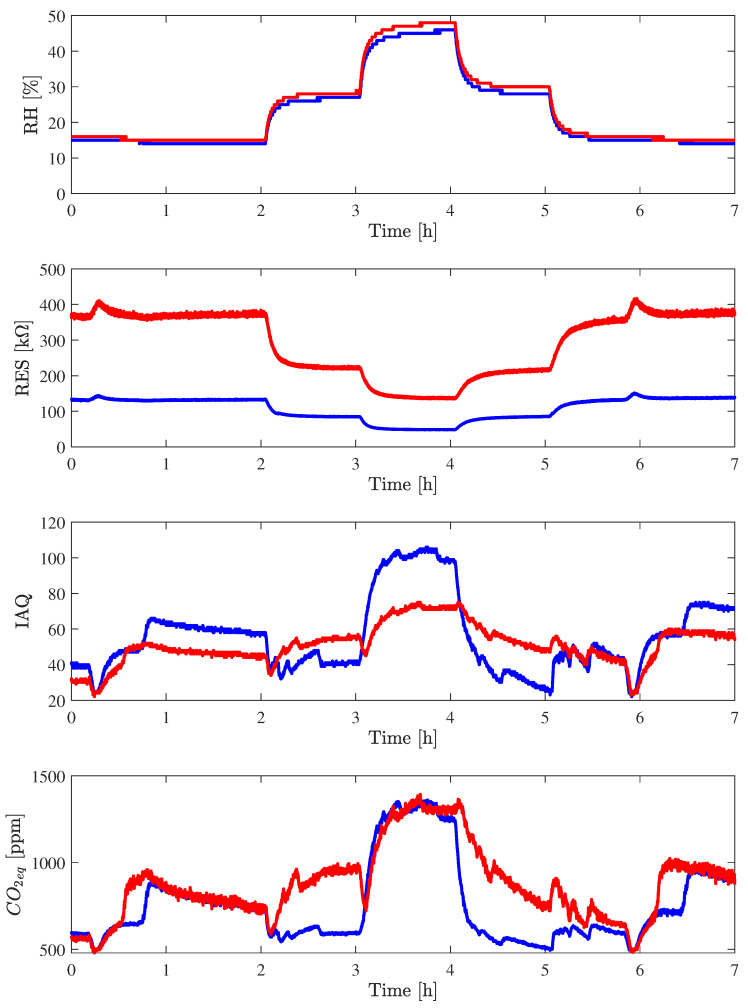
Behavior of RH, IAQ, CO_2eq_, and RES of Nicla_1_ (in blue) and Nicla_2_ (in red) at a temperature of 20 °C in the fluxing chamber.

The Pearson correlation matrix was computed starting from Sunday, 18 December 2023 at midnight to Friday 22 December 2023 at 13:45 p.m., in order to avoid the initial set-up period of the sensor. Values are reported in Table 8. Thanks to the controlled RH, the correlation between gas resistance and RH practically disappears; however, gas resistance is correlated with pressure, which is not controlled by the climatic chamber, and to the CO_2_, because the air is obtained from the laboratory in which the climatic chamber is located. Independently from fluctuations with a periodicity of 24 h of gas resistance and temperature, which are also visible in Figure 8, their correlation value is very low, suggesting an imperfect isolation of the chamber from the external environment.

### 6.3. Tests with CO_2_ and CO in the Fluxing Chamber

The purpose of the following measurements is to verify the use of the BME688 smart sensor outdoors, mounted on fully sustainable e-bike charging stations, for which the device was chosen based on its low-power characteristics. The first test was to check whether, as relative humidity increased, the IAQ indicator and CO_2_ estimation were still reliable and remained within acceptable limits.

Considering the fluxing chamber described in the previous section, two Nicla Sense ME boards, Nicla_1_ and Nicla_2_, were placed in the chamber, and the air was flushed at a temperature of 20 °C and relative humidity of 20% for 2 h in order to stabilize the readings of T, RH, and RES and the estimations of CO_2_ and IAQ made by BME688.

In Figure 8, the measurements made by Nicla_1_ and Nicla_2_ are reported. The collected measurements follow the test changes in relative humidity. In fact, as it is possible to observe, after the first 2 h, the relative humidity was raised to 40% for 1 h, then it was raised to 70% for the successive hour, then set again to 40% for 1 h, and finally adjusted again to 20% for 2 h, for a total of about 7 h of experiment. Even if the RH sensor was never calibrated, the measurements from the two devices are consistent, and a good agreement between the Nicla_1_ and Nicla_2_ is visible in the reported figure. The pressure measurements remain very stable during the entire test duration, with a standard deviation of 0.1 mbar computed over the 7 h of the experiment. The measurement of the raw gas resistance value differs between the two boards, but the trend is very similar, with a correlation coefficient between Nicla_1_ and Nicla_2_ of 0.99, calculated after the first half-hour to avoid the initial transient to the end of the experiment. It is worth noting how, for both Nicla, the falling time of RES, corresponding to the increase in RH, equal to about 10 min, is smaller than the rise time, equal to about 16 min, indicating how it is more complex to evaporate moisture with low-power thermal cycles. In addition, it is possible to see two peaks in the readings of gas resistance for both Niclas; this is due to glitches in the power supply interrupting the heating of the sensors.

Table 9 shows the cross-correlation matrix considering the central five hours only. This table reports a very high value of correlation between CO_2_ and IAQ, of the same order as the correlation between gas resistance and relative humidity. There is also a high correlation between CO_2_ and RH and between IAQ and RH, as expected. The high correlation between T and RH depends on the fluxing chamber, although the overall variation of temperature is within 0.2 °C during the 7 h of experiment.

The relative humidity cycle previously described was repeated at different temperatures: T = 10, 20, and 30 °C. Table 10 shows the mean values of gas resistance, CO_2_, and IAQ at different relative humidities and different temperatures measured by the Nicla_1_ subject to the test within the fluxing chamber. As for the previous tests, the mean values were computed only on the last 30 min of each cycle (e.g., referring to Figure 8, the mean value for T = 20 °C and RH = 20% refers to the mean value from hour 1:30 to hour 2:00 and from 5:30 to hour 6:00; for T = 20 °C and RH = 40%, the mean value was computed from hour 2:30 to hour 3:00 and from hour 4:30 to 5:00; and for T = 20 °C and RH = 70%, the mean value was calculated from hour 3:30 to hour 4:00). From the results reported, it is possible to note that gas resistance strongly depends on temperature and relative humidity, varying by one order of magnitude from T = 20 °C and RH = 20% to T = 20 °C and RH = 70%. The CO_2_ readouts remain within the standard deviation value, about 50 ppm if RH is below the 40%, but it sensibly varies when relative humidity reaches 70%. This CO_2_ estimation variation increases according to the temperature and, for T = 30 °C, it is three times the value related to RH = 40%. The IAQ readout sensibly increases only with RH = 70%, but the high humidity worsens the perceived air quality both with low temperature and with high temperature, according to human perception; the best value is with T = 20 °C with a standard deviation of about 10 a.u.

**Figure 9:**
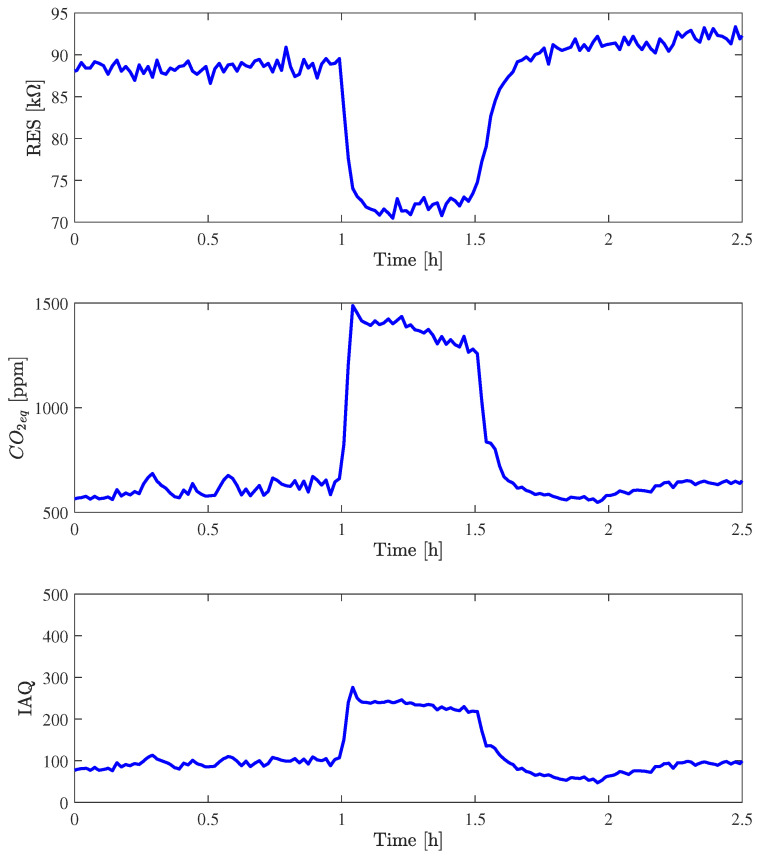
Behavior of gas resistance, CO_2eq_, and IAQ of Nicla_1_ at T = 20 °C and RH = 20% in the fluxing chamber with an injection of CO_2_ with 600 ppm concentration.

In order to verify the Nicla output in presence of a controlled flux of CO_2_ gas, 600 ppm of CO_2_ was injected for 30 min at a temperature of 20 °C with a relative humidity of 20%. Figure 9 shows the response, in terms of GAS resistance, CO_2_, and IAQ, to the injection and one hour before and one hour after. The mean value of estimated CO_2_, from hour 1:15 to hour 1:30, is 1332.7 ppm, which is quite different from the real value of 600 ppm. It should be noted that the fluxing chamber mixes dried purified air, with a concentration of CO_2_ of around 400 ppm, with CO_2_ from a bottle, and the 600 ppm is the gap between before and after the injection. If we compute the gap between the mean value of estimated CO_2_ fifteen minutes before the injection and the mean value from hour 1:15 and hour 1:30, it is equal to 616.4 ppm, which is quite similar to 600 ppm. Concerning the value of the IAQ, which increases from about 100 to about 240, it shows the same behavior analyzed with the test in the office: the value of 250 is quite simple to reach even with a variation of temperature or humidity, while values higher than 250 are related to thousands of parts per million of CO_2_.

**Figure 10:**
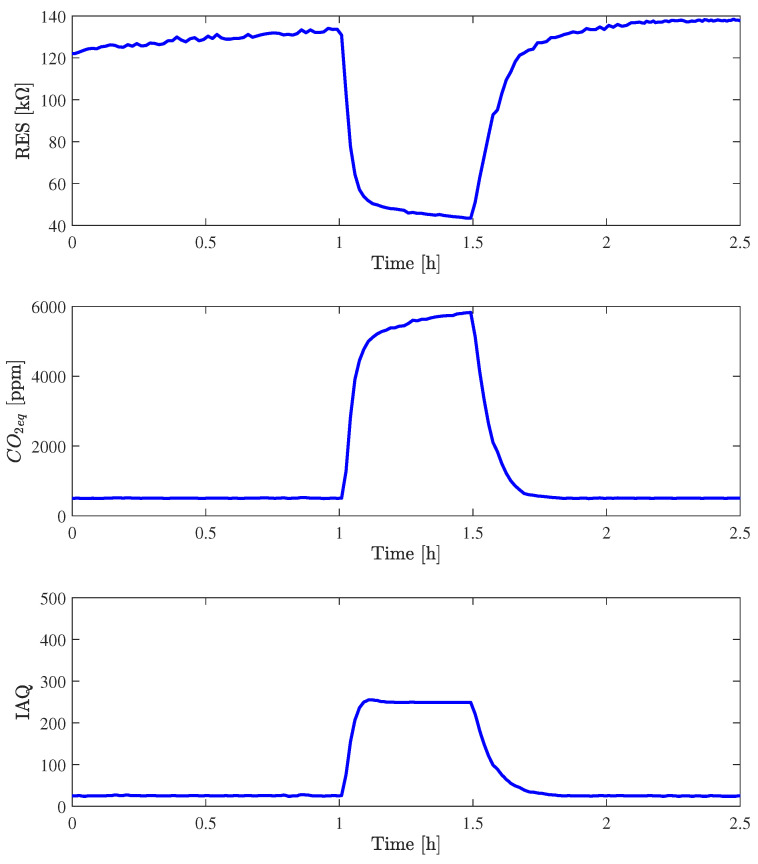
Behavior of gas resistance, CO_2eq_, and IAQ of Nicla_1_ at T = 20 °C and RH = 20% in the fluxing chamber with an injection of CO with 20 ppm concentration.

An important pollutant to be estimated in outdoor monitoring is CO, especially to detect wildfire events, as already demonstrated in [52]. In order to verify the Nicla response in the presence of this pollutant, a controlled flux of CO with a concentration of 20 ppm was injected for 30 min at a temperature of 20 °C and a relative humidity of 20%. Figure 10 shows the response, in terms of gas resistance, CO_2_, and IAQ, to this gas injection with a buffer period, in which no injection was present, of one hour before and one hour after. It should be noted that the baseline value of gas resistance is different from the value during the CO_2_ injection, but this is quite normal because the measurements were collected 12 days apart and the gas resistance value was affected by drift, a known issue of metal oxide gas sensors [53]. The gas resistance variation is negative in both injections; however, it is much more evident in presence of CO than with CO_2_. Comparing the two results, it is possible to note that, in the presence of CO at 20 ppm, the gas resistance decreases by about 80 kΩ, while with the injection of CO_2_ at 600 ppm, it varies by about 20 kΩ. This result, indicates a very good sensitivity with respect to CO. The CO_2_ readout is higher than 5000 ppm, a value that has a similar negative effect on persons; exposure limits recommendations previously reported (e.g., ECHA) are almost the same for 5000 ppm of CO_2_ and 20 ppm of CO. The IAQ readout remains below the 250 threshold, although the tests in the office show that in the presence of 5000 ppm of CO_2_ or more, the IAQ exceeds 300.

### 6.4. Results Comparison

In order to further investigate the previously highlighted correlation, the statistical dependence between the rankings of IAQ and the other measures obtained from the BME688 was evaluated. In Table 11, the PCC is compared against Spearman’s rank correlation coefficient (SRCC), calculated as follows:

(2) $$r_{xy}=\frac{\mathrm{cov}(R(x),R(y))}{\sigma_{R(x)}\sigma_{R(y)}},$$

where R(x) and R(y) are the rank variables relative to the *x* and *y* measurements, cov is the covariance operator, and $\sigma_{R(x)}$ and $\sigma_{R(y)}$ are the standard deviations of the two rank variables. In the table, the PCC and SRCC are calculated in three different scenarios:

- Studio setup: consisting of the overall acquisition in the experimental setup previously described, where temperature and humidity are not directly controlled;
- Fluxing chamber: consisting of the acquisitions made at a constant temperature of 20 °C in the fluxing chamber with a varying relative humidity as previously reported;
- Climatic chamber: consisting of the measurements gathered in the climatic chamber setup where both temperature and relative humidity are maintained constant.

**Table 11:** Pearson correlation coefficient (PCC) and Spearman’s rank correlation coefficient (SRCC) for IAQ in different experimental tests.

	Studio Setup				Fluxing Chamber				Climatic Chamber	
	Nicla_1_		Nicla_2_		Nicla_1_		Nicla_2_		Nicla_3_	
	PCC	SRCC	PCC	SRCC	PCC	SRCC	PCC	SRCC	PCC	SRCC
T	0.125	0.037	0.169	0.088	0.229	0.096	0.417	0.519	−0.065	−0.114
RH	−0.127	−0.120	−0.204	−0.194	0.635	0.175	0.858	0.837	−0.014	0.018
P	0.253	0.260	0.256	0.268	0.149	0.359	−0.096	−0.073	−0.169	−0.196
RES	−0.108	−0.092	−0.005	−0.004	−0.517	−0.258	−0.840	−0.873	−0.013	0.035
CO_2eq_	0.666	0.924	0.660	0.926	0.989	0.997	0.983	0.971	0.976	0.998

As an immediate result, it is possible to confirm the correlation between the IAQ and the CO_2eq_ estimation; this is confirmed by the minimum calculated SRCC value of 0.924 in all the reported experiments. The scarce direct dependence of the IAQ estimation is also evident from the measured variables (T, RH, P, and RES); in fact, both PCC and SRCC are, in most of the cases, lower than 0.3. In the case of fluxing chamber results, where the humidity was cycled, the IAQ has a high correlation with the measured gas resistance and the relative humidity; in this case, SRCC reports an inverse correlation between the the IAQ and RES variables with a minimum value of 0.25. When the temperature and humidity are under the control of the climatic chamber, the dependence of the IAQ from temperature, relative humidity, and pressure is limited to 0.2; meanwhile, its correlation with the gas resistance is almost negligible.

## 7. Conclusions

Air quality monitoring is a key topic for people riding bikes and e-bikes. In this paper, an air quality sensor designed for HVAC was tested to be used as an outdoor low-power, low-cost sensors to indicate the perceived air quality, preventing health risks. The Nicla Sense ME, based on the BME688 sensor by Bosch, was used with a special attention given to raw values, to the estimated CO_2_ equivalent, and the index for air quality IAQ. Three setups were designed to test the sensor behavior indoors: in a non-controlled environment, in a climatic chamber controlling temperature and relative humidity, and in a 1 L fluxing chamber controlling temperature, relative humidity, and gas.

According to the measurements made in the climatic chamber setup, experimental results show that temperature, humidity, and pressure readouts are stable and precise. On the other hand, as also suggested by the manufacturer, the sensor has a good sensitivity with respect to CO_2_ and CO, as was demonstrated with the readings of the gas resistance at different concentrations of the two gasses in the tests carried out in the fluxing chamber. The CO_2_ equivalent is a good parameter indicating the pollution perceived by humans; the experiments showed how 20 ppm of CO was considered equivalent to 5000 ppm of CO_2_ as a threshold of attention.

Compared to the direct measurement and to the CO_2_ estimation made by the sensor, the IAQ value is not a reliable indication for assessing the overall perceived air quality in the outdoor environment. As was shown in the experiments, the IAQ value remained below the threshold of 250 for variations of temperature, humidity, and for CO injections, while it exceeded this threshold indoors and in crowed rooms. The BME688 built-in software appeared to compensate for the effect of temperature and relative humidity on CO_2_ and IAQ estimates, as shown in the experiments carried out in the fluxing chamber. However, in the presence of large relative humidity (e.g., T = 30 °C and RH = 70%), an overestimate of CO_2_ (more than 1000 ppm) was detected. Therefore, in order to use this sensor for outdoor applications where it is plausible to reach high percentages of humidity, additional compensation mechanisms should be designed.

Future work could exploit heating profile modulation supported by BME688 with respect to the environmental conditions and the expected pollutants. Moreover, additional experiments should be carried out in an outdoor environment where humidity and temperature conditions can not be controlled and maintained like in the thermal chamber and fluxing chamber presented in this work. Multi-sensor platforms and combinations of different sensing technologies in conjunction with ad hoc constructed AI models could also be investigated. 

## Figures and Tables

**Table 1:** Regulatory and recommended limits for some pollutants. The meanings of abbreviations are reported in the section on abbreviations.

Substance	EC No.	CAS No.	Regulatory Limits		Recommended Limits		
			OSHA PEL [16,17]	Cal/OSHA PEL [16,17]	NIOSH REL [16,17]	ACGIH TLV [16,17]	ECHA-OELs [18,19,20,21]
			8-h TWA [ppm]	8-h TEA [ppm]	Up to 10-h TWA [ppm]	8-h TWA [ppm]	8-h TWA [ppm]
Carbon monoxide CO	211-128-3	630-08-0	50	25 (C) 200	35 (C) 200	25	20 (ST) 100 (IV)
Carbon dioxide CO_2_	204-696-9	124-38-9	5000	5000 (ST) 30,000	5000 (ST) 30,000	5000 (ST) 30,000	5000 (II)
Nitrogen dioxide NO_2_	233-272-6	10102-44-0	(C) 5	(ST) 1	(ST) 1	0.2	0.5 (ST) 1 (IV)
Hydrogen ulphide H_2_S	231-977-3	7783-06-4	(C) 20 (C) 50 *	10 (ST) 15 (C) 50	(C) 10 [10 min]	1 (ST) 5	5 (ST) 10 (III)
Ethanol CH_3_CH_2_OH	200-578-6	64-17-5					1000 ** (ST) 5000 **

* Acceptable maximum peak above the acceptable ceiling concentration for an 8 h shift, maximum duration: 10 min once only if no other measurable exposure occurs. ** France: Tableaux des maladies professionnelles Régime général tableau 84.

**Table 2:** Brief comparison between commercially available sensors for air quality monitoring.

Device	Manufacturer	Technology	Target	Accuracy (If Applicable) [ppm]	Power Consumption [mW]	Cost [EUR/kunit]
PASCO2V01	Infineon	PAS	CO_2_	30	30	∼5
TGS5141-P00	Figaro	MOx	CO	–	–	∼5
AQ7CO	Honeywell	MOx	CO_2_	up to 0.01	–	25.99
ENS160	ScioSense	MOx	TVOC	–	58	4.6
ZMOD4410	Renesas	MOx	TVOC	30	1.5–23 *	1.82
SGP30	Sensirion	MOx	PM, TVOC, NOx	1	∼90	17
BME680	Bosch Sensortec	MOx	TVOC (qualitative)	–	0.18–24 *	4.75
BME688	Bosch Sensortec	MOx	TVOC (qualitative)	–	0.18–7.8 *	4.75
CCS811	Bosch Sensortec	MOx	TVOC	–	60	4.75

* Depending on the operation mode set on the sensor.

**Table 3:** BSEC IAQ index interpretation. Taken from [35].

IAQ Index	Air Quality	Impact (Long-Term Exposure)	Suggested Action
0–50	Excellent	Pure air; best for well-being	No measures needed
51–100	Good	No irritation or impact on well-being	No measures needed
101–150	Lightly polluted	Reduction of well-being possible	Ventilation suggested
151–200	Moderately polluted	More significant irritation possible	Optimize ventilation
201–250	Heavily polluted	Exposition might lead to effects like headache depending on type of VOCs	Optimize ventilation
251–350	Severely polluted	More severe health issue possible if harmful VOC present	Contamination should be identified if level is reached even w/o presence of people; maximize ventilation and reduce attendance
>351	Extremely polluted	Headaches, additional neurotoxic effects possible	Contamination needs to be identified; avoid presence in room and maximize ventilation

**Table 4:** Statistics of raw values of T, RH, and P from Nicla_1_ and Nicla_2_.

Nicla	T			RH			P		
	[°C]			[%]			[mbar]		
	μ	σ	Δ	μ	σ	Δ	μ	σ	Δ
1	23.7	4.7	19.7	26.2	8.5	21.0	995	10	37
2	23.7	4.7	20.9	25.7	8.5	22.0	995	10	37

**Table 5:** Pearson correlation matrix with office empty and not air-conditioned for Nicla_1_.

$\rho_{\mathrm{xy}}$		*x*				
		RH	P	RES	CO_2eq_	IAQ
*y*	T	−0.473	0.263	−0.761	0.063	0.051
	RH	1.000	−0.855	0.312	−0.210	−0.203
	P		1.000	−0.366	0.400	0.398
	RES			1.000	−0.512	−0.517
	CO_2eq_				1.000	0.974

**Table 6:** Pearson correlation matrix with office empty and air-conditioned for Nicla_1_.

$\rho_{\mathrm{xy}}$		*x*				
		RH	P	RES	CO_2eq_	IAQ
*y*	T	0.296	−0.417	−0.271	−0.313	−0.331
	RH	1.000	−0.846	−0.803	−0.489	−0.615
	P		1.000	0.687	0.662	0.751
	RES			1.000	0.233	0.413
	CO_2eq_				1.000	0.932

**Table 7:** Pearson correlation with office air-conditioned and occupied for Nicla_1_.

$\rho_{\mathrm{xy}}$		*x*				
		RH	P	RES	CO_2eq_	IAQ
*y*	T	0.186	−0.267	−0.229	0.193	0.091
	RH	1.000	−0.813	−0.357	0.009	−0.116
	P		1.000	0.474	−0.149	−0.027
	RES			1.000	−0.878	−0.735
	CO_2eq_				1.000	0.891

**Table 8:** Pearson correlation matrix within the climatic chamber office for Nicla_3_.

$\rho_{\mathrm{xy}}$		*x*				
		RH	P	RES	CO_2eq_	IAQ
*y*	T	−0.049	−0.091	0.021	0.064	0.127
	RH	1.000	−0.116	0.092	−0.131	−0.172
	P		1.000	−0.616	0.073	0.135
	RES			1.000	−0.709	−0.761
	CO_2eq_				1.000	0.975

**Table 9:** Pearson correlation matrix within the fluxing chamber during RH cycle for Nicla_3_.

$\rho_{\mathrm{xy}}$		*x*				
		RH	P	RES	CO_2eq_	IAQ
*y*	T	0.708	−0.397	−0.703	0.277	0.229
	RH	1.000	−0.235	−0.978	0.690	0.635
	P		1.000	0.221	0.113	0.149
	RES			1.000	−0.568	−0.517
	CO_2eq_				1.000	0.989

**Table 10:** Mean values of resistance (RES), estimated CO_2_ (CO_2eq_), and IAQ within the fluxing chamber for the Nicla_1_.

		RES [kΩ]			CO_2eq_ [ppm]			IAQ		
	RH	20%	70%	70%	20%	70%	70%	20%	70%	70%
T										
10 °C		228.5	157.6	102.5	644.2	618.1	906.4	96.4	89.8	174.9
20 °C		133.3	84.3	48.7	674.4	573.2	1321.1	49.9	37.7	102.5
30 °C		84.1	51.1	27.0	581.1	629.9	1968.4	65.1	80.8	243.1

## Data Availability

Data are contained within the article.

## Abbreviations

The following is a list of abbreviations used in Table 1:

OSHA	Occupational Safety and Health Administration
Cal/OSHA	California Division of Occupational Safety and Health Administration
NIOSH	National Institute for Occupational Safety and Health
ACGIH	American Conference of Governmental Industrial Hygienists
ECHA	European Chemicals Agency
PEL	Permissible Exposure Level
REL	Recommended Exposure Level
OELs	Occupational Exposure Limits
ST	STEL—Short Term Exposure Limit
C	Ceiling
TWA	Time-Weighted Average
